# Epigenetics in Prostate Cancer

**DOI:** 10.1155/2011/580318

**Published:** 2011-11-30

**Authors:** Costantine Albany, Ajjai S. Alva, Ana M. Aparicio, Rakesh Singal, Sarvari Yellapragada, Guru Sonpavde, Noah M. Hahn

**Affiliations:** ^1^Indiana University Melvin and Bren Simon Cancer Center, Indianapolis, Indiana 46202, USA; ^2^Veterans Affairs Medical Center and the Baylor College of Medicine, Houston, TX 77030, USA; ^3^Department of Genitourinary Medical Oncology, M.D. Anderson Cancer Center, Houston, TX 77030, USA; ^4^University of Miami Sylvester Comprehensive Cancer Center, Miami, FL 33136, USA; ^5^Texas Oncology and U.S. Oncology Research, Houston, TX 77060, USA

## Abstract

Prostate cancer (PC) is the most commonly diagnosed nonskin malignancy and the second most common cause of cancer death among men in the United States. Epigenetics is the study of heritable changes in gene expression caused by mechanisms other than changes in the underlying DNA sequences. Two common epigenetic mechanisms, DNA methylation and histone modification, have demonstrated critical roles in prostate cancer growth and metastasis. DNA hypermethylation of cytosine-guanine (CpG) rich sequence islands within gene promoter regions is widespread during neoplastic transformation of prostate cells, suggesting that treatment-induced restoration of a “normal” epigenome could be clinically beneficial. Histone modification leads to altered tumor gene function by changing chromosome structure and the level of gene transcription. The reversibility of epigenetic aberrations and restoration of tumor suppression gene function have made them attractive targets for prostate cancer treatment with modulators that demethylate DNA and inhibit histone deacetylases.

## 1. Introduction

Unlike mutations which cause permanent changes in DNA sequence, epigenetic changes do not alter the coding sequence of genes. They induce conformational changes in the DNA double helix and modify access of transcription factors to promoter regions upstream of coding sequences [1]. The epigenome comprises a tissue-specific profile of DNA methylation, histone modifications, nucleosome remodeling, and RNA-associated silencing. Cancer is a disease driven by progressive genetic and epigenetic aberrations that manifest as global alterations in chromatin packaging and by specific promoter changes that influence the transcription of associated genes [1, 2]. In the carcinogenesis of prostate cancer, somatic epigenetic alterations appear earlier and more frequently than genetic sequence changes. Multiple genes functionally silenced by epigenetic alterations have been identified, providing new molecular biomarkers of prostate cancer and new mechanistic clues into prostate cancer etiology [3]. This paper will focus on the preclinical evidence implicating the epigenome as a key mediator in prostate carcinogenesis and summarize initial clinical trial experiences with epigenetic targeted agents.

## 2. Review Criteria

We searched the PubMed database for articles with the terms “prostate cancer”, “epigenetics”, “hypermethylation”, “hypomethylation”, “histone acetylation”, “HDAC”, and “DNMT”. Original full-text articles published in English were reviewed. The reference lists of identified articles were searched for further relevant papers. No limits were set on the years of publication. To limit the number of references, throughout this paper, we have cited reviews rather than original articles when dealing with matters that are well established or of a more general nature.

## 3. DNA Methylation

**Figure 1:**
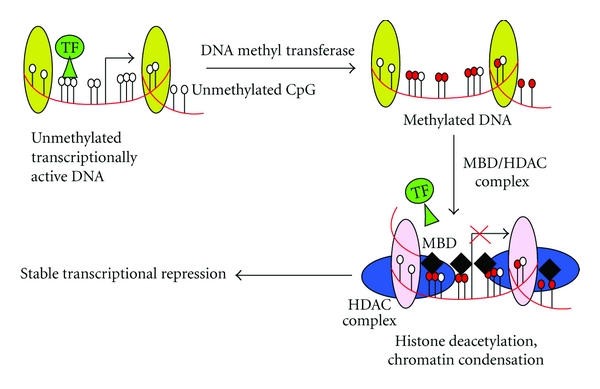
Epigenetic mechanism of gene expression silencing. (a) In unmethylated DNA (depicted by white hollow circles on left) transcription factors (TF) are free to bind gene promotor regions. In hypermethylated DNA (depicted in red filled-in circles on the right) TF are blocked from binding to gene promotor regions leading to functional silencing of gene expression. (b) Histone deacetylation by methyl-CpG-binding domain protein (MPD)/histone deacetylase (HDAC) complexes promotes a condensed structure which inhibit normal gene transcription. (With permission from [4].)

DNA methylation is an important regulator of gene transcription, and its role in carcinogenesis has been a topic of considerable interest in the last few years. Hypermethylation represses transcription of CpG-rich promoter regions of tumor suppressor genes leading to gene silencing. DNA methylation is a covalent chemical modification, resulting in the addition of a methyl (-CH_3_) group at the carbon-5 position of the cytosine ring. This reaction is catalyzed by DNA methyltransferase (DNMT) in the context of the sequence 5′-CG-3′ (also called the CpG dinucleotide) [5]. CpGs are nonrandomly distributed, and around 1% of human DNA consists of short, CpG-dense sequences termed CpG islands [6, 7]. In the unmethylated state, chromatin at these CpG island regions can be molded into active conformations that can facilitate the loading of RNA polymerases onto gene promoters. However, 60–90% of CpG dinucleotides are methylated in the adult genome, and this modification results in the spontaneous deamination of 5-methylcytosine to thymine; this reaction changes the chromatin structure and poses a significant barrier to transcription [7] (Figure 1(a)). Approximately half of all the genes in humans have CpG islands, and these are present on both housekeeping genes and genes with tissue-specifc patterns of expression [8]. Promoter region CpG islands are usually unmethylated in all normal tissues, regardless of the transcriptional activity of the gene. The main exceptions include nontranscribed genes on the inactive X-chromosome and imprinted autosomal genes where one of the parental alleles may be methylated [9]. 

Three active DNA methyltransferases have been identified (*DNMT1*, *DNMT3A*, and *DNMT3B*). *DNMT1* is principally responsible for maintenance of the cell methylation profile and to less extent *de novo *methylation of tumor suppressor genes. The *de novo* activity of *DNMT1* has been shown to be stimulated by aberrant DNA structures [10]. *DNMT3A* and *DNMT3B* have both maintenance and *de novo* methylation activities and are believed to be responsible for the wave of methylation that occurs during embryogenesis [2]. Recently, growing evidence has, however, indicated that the DNA methylation machinery is in fact more complicated. For example, it has been demonstrated that DNMTs physically bind to several histone modifiers including histone deacetylases (HDACs) and EZH2. The formation of multicomponent epigenetic regulatory complex suggests that DNA methylation and histone modification machineries function in a highly cooperative manner in regulating chromatin structure and gene expression [11].

## 4. Hypermethylation in Prostate Cancer

Prostate cancer cells commonly have promoter hypermethylation as a means of gene repression in the acquisition and maintenance of the neoplastic phenotype. This modification silences many classic tumor-suppressor gene functions including hormone signaling, DNA repair, cell adhesion, cell-cycle control, and apoptosis [12–14]. Specific genes implicated within each category are summarized in Table 1. Tumor suppressor genes frequently altered in other human cancers such as *PTEN*, *RB1*, and *TP53* are not commonly hypermethylated in PC, although allelic loss and point mutations are observed in advanced stage cases [15].

### 4.1. Hormone Signaling

By far the most studied transcriptional activator in prostate cancer is the androgen receptor (AR). The AR is a nuclear hormone receptor, which is activated by binding of androgen ligands. The AR is a critical effector of prostate cancer development and progression. Since the growth of PC is initially androgen sensitive, metastatic disease has been traditionally treated by androgen deprivation therapy (ADT). Despite an initial disease control, progression uniformly occurs due to emergence of castration-resistant PC cells. Recent studies demonstrated the continued role of the AR in driving PC cell growth even in the presence of low levels of circulating androgens and the emergence of a castrate-resistant prostate cancer (CRPC) phenotype [16–18]. Epigenetic changes including CpG methylation and histone acetylation play important roles in the regulation of AR pathway signaling [19]. Hypermethylation of the AR gene (*AR*) is more frequent in CRPC tissues (29%) compared with untreated primary tissues (10%) suggesting that hypermethylation may contribute to the development of a castrate-resistant phenotype [19, 20]. 

In preclinical studies with prostate cancer cells, Gravina et al. investigated the potential reversibility of castrate resistance in PC cell lines (the AR positive 22RV1 and the AR negative PC3) with the hypomethylating agent azacitidine in combination with the antiandrogen bicalutamide [21, 22]. The addition of azacitidine to bicalutamide induced apoptosis in both cell lines and was associated with upregulation of several proapoptotic mediators (e.g., p16, Bax, Bak, and p21) with corresponding downregulation of antiapoptotic factors (e.g., Bcl-2 and Bcl-XL). Interestingly, in PC3 cells, the AR gene (*AR)* was reexpressed and correlated with response to combination therapy. However, *AR* expression did not correlate with response in the 22RV1 *AR* positive cell line suggesting a “necessary but not sufficient” need to express *AR* for activity of hypomethylating agents in this model [23]. Another study investigated hypomethylation as a therapeutic option to counteract resistance to androgen deprivation in both *AR* positive (LNCaP-HR and 22RV1-HR) and negative cell lines (PC-3) [24]. Inhibition of DNA methylation reversed castrate resistance correlating with decreased *DNMT1*-dependent *STAT3* activity.

Of note, not only the AR, but also other members of the steroid hormone receptor superfamily may play a role in normal prostate function and tumorigenesis. For instance, the *ESR1* and *ESR2* genes encoding the estrogen receptors, ER*α* and ER**β**, are hypermethylated at low frequencies in PC [15].

### 4.2. DNA Repair Genes

One of the earliest changes in the pathogenesis of prostate cancer is CpG island hypermethylation at the glutathione S-transferase (*GSTP1*) gene. *GSTP1* is involved in the metabolism, detoxifcation, and elimination of potentially genotoxic foreign compounds and thus acts to protect cells from DNA damage and cancer initiation. The CpG island promoter region spanning *GSTP1* gene becomes methylated in the majority of prostate tumors. The gene is expressed and unmethylated in all normal tissues [25]. No mutations or deletions have been reported for *GSTP1* gene in prostate cancer; however the gene is inactivated and both alleles are commonly methylated [26]. Promoter methylation of *GSTP1 *is absent in normal epithelium and present in 6.4% of proliferative inflammatory atrophy, in 70% of high-grade prostatic intraepithelial neoplasia and in 90% of prostate cancer [27]. The *GSTP1* gene encodes the *π*-class glutathione *S*-transferase (GST), an enzyme capable of detoxifying electrophilic and oxidant carcinogens [28]. The associated loss of *π*-class GST function likely sensitizes prostatic epithelial cells to cell and genome damage inflicted by dietary carcinogens and inflammatory oxidants, perhaps explaining the well-documented contribution of diet and lifestyle factor to prostatic carcinogenesis [16]. *GSTP1* methylation appears to discriminate between benign and premalignant/malignant prostate and persists through all stages of prostate cancer, and can be detected in circulating tumor cells (CTCs) [29–32]. 

The DNA repair protein methylguanine DNA methyltransferase (*MGMT*) removes alkyl adducts from the O^6^ position of guanine. *MGMT* expression is decreased in some tumor tissues and in cell lines. Loss of expression is rarely due to deletion, mutation, or rearrangement of the *MGMT* gene, but methylation of discrete regions of the CpG islands of *MGMT* has been associated with the silencing of the gene in cell lines [33]. *MGMT* hypermethylation plays an important role in development of prostate carcinoma. In one study the development of prostate carcinoma was correlated with the methylation pattern of MGMT [34].

### 4.3. Tumor Suppression Genes

Promoter methylation in *APC* has been identified as a marker for prostate cancer prognosis. Patients with methylation in *APC* had higher prostate cancer mortality than patients with an unmethylated cancer [35]. The *APC* complex is known from studies of colorectal cancer cells to function as a gatekeeper in the cell, preventing the transcription of gene products that promote cell proliferation and survival rather than differentiation and apoptosis [36]. Hypermethylation of *APC* implies silencing of this gatekeeper function, making the cell vulnerable to further epigenetic and genetic changes and, thus, progression toward invasive cancer. 

Retinoic acid receptor beta (*RAR*β**) and *PDLM4* have been shown to function as tumor suppressor genes in human prostate cancer cell and xenograft models. *RAR*β** and *PDLM4* promoters are commonly hypermethylated during prostate cancer progression [37, 38]. Retinoid acid (RA) exerts its biological affect through two families of nuclear receptors: RA receptors (RAR *α*, *β*, *γ*) and retinoid X receptors (RXR *α*, *β*, *γ*), which are ligand-dependent transcription factors of the steroid/thyroid hormone nuclear receptor superfamily. *RAR*β*2* is located in chromosomal region 3p24 and has been shown to harbor a CpG-rich region in its promoter [39], which is frequently hypermethylated in prostate cancer [14]. Jerónimo et al. showed *RAR*β*2* hypermethylation in 97.5% of PC, 94.7% of high-grade prostatic intraepithelial neoplasia (HGPIN), and 23.3% of BPH. Methylation levels were significantly higher in PC compared with HGPIN and BPH (*P* < 0.00001) [37].The tazarotene-induced gene 1 (TIG1), also known as RAR-responsive 1 gene, was first identified as an RA-responsive gene and was shown to be downregulated in prostate cancer. It is proposed that *RAR*β** silencing by promoter methylation is a crucial event in prostate tumor progression and that epigenetic changes in the *TIG1* promoter, and possibly in the promoters of other retinoid response genes, are downstream events to *RAR*β** deficiency. Thus, in the case of *TIG1*, silencing affects cell-cell contacts and results in increased proliferation and invasiveness of tumor cells [40]. 

In addition, inactivation of the tumor suppressor gene *RASSF1A* has been associated with hypermethylation of its CpG-island promoter region [14]. Selective promoter methylation of the *RASSF1A* promoter, but not of *RASSF1C*, is observed in 53% of prostate cancers and is associated with higher Gleason score and serum PSA [14]. The encoded RASSF1A protein was found to interact with DNA repair protein XPA. Furthermore, the RASSF1A protein has also been shown to counteract stimulation of cell proliferation by *RAS*-linked pathways and inhibit the accumulation of cyclin D1 and thus induce cell cycle arrest [41].

### 4.4. Cell Adhesion Genes

Invasion and metastasis are acquired properties during prostate cancer progression, and involve cancer cells losing intercellular contact, becoming motile, and invading surrounding tissues. E-cadherin (*CDH1*) is a strong suppressor of invasion. Decreased *CDH1* expression has been associated with more extensive metastases and poor overall survival in prostate cancer patients [42, 43]. The 5′ CpG island of *CDH1* is densely methylated in prostate cancer cell lines (DuPro, TSUPr1, and FNC) [44]. Increased hypermethylation of the *CDH1* promoter has been observed in association with fibroblastic cell morphology characteristic of epithelial-to-mesenchymal transition in nonprostate malignancies [45]. *CD44* encodes for another integral membrane protein involved in matrix adhesion and signal transduction. In prostate cancer, *CD44* hypermethylation is seen in 78% of patients compared to only 10% of patients without cancer [46, 47]. Thus, *CD44* may be another important mediator of prostate carcinogenesis.

### 4.5. Cell Cycle and Proapoptotic Genes

The protein encoded by *CCND2* gene belongs to the highly conserved cyclin family, whose members are characterized by a dramatic periodicity in protein abundance through the cell cycle. Cyclin D forms a complex with and functions as a regulatory subunit of CDK4 or CDK6, whose activity is required for cell cycle transition from G1 to S phase. Hypermethylation of the *CCND2* promoter is significantly higher in prostate cancers compared to normal prostate tissues (32%, 6% resp.; *P* = 0.004), and there are statistically significant concordances between methylation of *CCND2* and the methylation of *RAR*β**, *GSTP1*, *CDH13*, *RASSF1A*, and *APC* genes [48]. High *CCND2* methylation levels characterize invasive PC, correlating with clinicopathologic features of tumor aggressiveness [49].

*GADD45*α** (growth arrest and DNA damage inducible gene 45 a) is a tumor suppressor gene involved in maintenance of genomic stability, DNA repair, and cell-cycle control. It is thought to modulate DNMT1 activity at sites of repair of double-stranded DNA repair during homologous recombination [50]. *GADD45*α** partially mediates docetaxel cytotoxicity and can cause active hypomethylation of CpG residues without the need for DNA replication. *GADD45*α** is itself methylated at 4 CpG sites proximal to the promoter region in several epithelial cancers including prostate and breast cancer [23]. Preclinical work in prostate cancer cell lines has revealed increased methylation of *GADD45*α** in DU145 and LNCaP and decreased methylation in PC3 that correlated inversely with gene expression [51]. Enhanced sensitivity to docetaxel was observed by upregulation of *GADD45*α** in DU145 cells by recombinant expression of *GADD45*α** or pretreatment with 5-azacitidine.

TMS1 (Target of Methylation Induced Silencing 1), also known as ASC (Apoptosis Speck-like protein containing a CARD), is a proapoptotic gene that has been shown to play an important role in the progression of many cancers. TMS1 encodes a protein-containing pyrin domain (PYD) in the N-terminus and a caspase recruitment domain (CARD) in the C-terminus, both of which are members of the death domain-fold superfamily. It is believed that TMS1 induces apoptosis via the caspase-9 pathway 10 [52, 53]. Methylation of TMS1 is a frequent event in prostate cancer, and loss of TMS1/ASC gene expression is associated with complete methylation of the promoter region in LNCaP prostate cancer cells [54].

## 5. Hypomethylation in Prostate Cancer

Hypomethylation is a second methylation defect that is observed in a wide variety of malignancies including prostate cancer [55]. Hypermethylation changes seem to precede hypomethylation changes, which are generally detected in cancers of higher stage and histologic grade and occur heterogeneously during prostate cancer progression and metastatic dissemination [56, 57]. Hypomethylation is observed due to the diminished methylation of abundant repetitive sequences that are densely methylated in normal cells, such as LINE-1 retrotransposons [58]. Hypomethylation has been hypothesized to contribute to oncogenesis through multiple mechanisms including: activation of oncogenes such as *c-MYC* and *H-RAS*, activation of latent retrotransposons, and by contributing to chromosome instability [5]. Recent studies have demonstrated strong association between *MYC* overexpression in prostate cancer tissues and clinical progression [59]. *MYC* is required for androgen-dependent growth and following its ectopic expression can induce androgen-independent growth in prostate cancer cells [60].

The *PLAU* gene is highly expressed in most prostate cancer tissues and invasive prostate cancer cell lines [61, 62]. The *PLAU* gene encodes urokinase plasminogen activator, a multifunctional protein that can promote tumor invasion and metastasis in several malignancies including prostate cancer [3].

DNA hypomethylation has been associated with increased rates of genomic instability. Specifically, there is a strong association between alterations on chromosome 8 and genome-wide hypomethylation. This association suggests that *PLAU* hypomethylation and alterations in chromosome 8 may be mechanistically linked to each other in prostate carcinoma [63].

### 5.1. Histone Modification

Three key regulators of histone modification are histone deacetylases (HDACs), histone acetyltransferases (HAT), and histone methyltransferases [64, 65]. Together, HDACs and HATs determine the acetylation status of histones. Histones are no longer considered to be simple “DNA-packaging” proteins; they are recognized as being dynamic regulators of gene activity that undergo many posttranslational chemical modifications, including acetylation, methylation, and phosphorylation. The N-terminal tails of histone proteins, which protrude out of the nucleosome, are rich in positively charged amino acids that are subject to various reversible posttranslational modifications. The status of acetylation and methylation of specific lysine residues contained within the tails of nucleosomal core histones is known to have a crucial role in regulating chromatin structure and gene expression [66]. Histone modifications, together with DNA methylation, also have a vital role in organizing nuclear architecture [64], which, in turn, is involved in regulating transcription and other nuclear processes. Alterations of histone modification patterns have the potential to affect the structure and integrity of the genome and to disrupt normal patterns of gene expression, which could be causal factors in cancer [66]. 

Histone acetylation mediated by HATs is correlated with transcriptional activation, and histone deacetylation mediated by HDACs is linked to gene silencing (Figure 1(b)). By removal of acetyl groups from histones, HDACs create a nonpermissive chromatin conformation that prevents the transcription of genes that encode proteins involved in tumorigenesis. Histone methylation on arginine and lysine can be associated with either gene activation or suppression depending on the amino acid position and the number of methylated residues [67, 68]. Polycomb proteins form chromatin-modifying complexes that implement transcriptional silencing in higher eukaryotes. Hundreds of genes are silenced by Polycomb proteins, including dozens of genes that encode crucial developmental regulators in organisms ranging from plants to humans. Two main families of complexes, called Polycomb repressive complex 1 (PRC1) and PRC2, are targeted to repressed regions.

### 5.2. Histone Modification in Prostate Cancer

 In PC cell lines methylation of lysine 9 in histone 3 (*H3K9*) is linked to repression of AR genes [69], and histone *H3K4* methylation is associated with AR gene activation in CRPC cell lines and tissues [70]. *H3K4* is significantly methylated at the AR enhancer of the protooncogene *UBE2C* gene in CRPC, which leads to AR binding and *UBE2C* gene expression [70, 71]. Heat shock protein 90 (*TRAP1*) plays a key role in androgen-induced and -independent nuclear localization and activation of AR. Histone deacetylase 6 (*HDAC6*) regulates AR hypersensitivity and nuclear localization, mainly via modulating *TRAP1* acetylation [72].

Upregulation of two AR coactivators potently increases cellular androgen sensitivity. Some of the best studied AR coactivators are members of the family of *SRC1* and transcriptional intermediary factor 2 (*TIF2*) [73, 74]. The proteins encoded by *SRC1* and *TIF2* possess histone acetylase activities, but are also able to recruit other histone acetylases such as the *CREB*-binding protein p300 and *PCAF* [75]. An analysis of prostate cancer samples from patients, who failed endocrine therapy, showed that expression of *SRC1* and *TIF2* was more intense than in those from patients with benign prostatic hyperplasia or androgen-dependent tumors [73]. 

Increasing evidence suggests that histone modification plays important role during prostate tumorigenesis. Changes in global levels of individual histone modifications are predictive of the clinical outcome of prostate cancer independently of other features such as tumor stage, preoperative prostate-specific antigen levels, and capsule invasion [76], and may help to identify patients with adverse prognosis and high risk for recurrence [77, 78]. Specifically, global methylations of *H3K4* and histone H3 lysine 18 acetylation (*H3K18Ac*) are independent predictor of recurrence in low-grade prostate cancer [76, 79].

**Figure 2:**
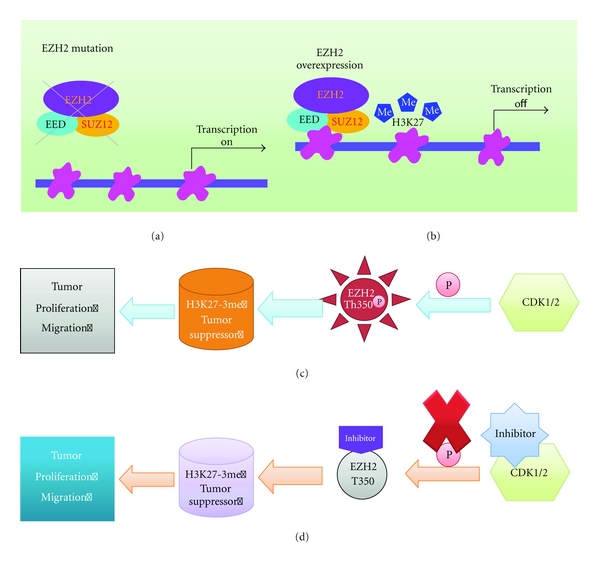
(a) The mutation of EZH2 usually turns on gene transcription. (b) Overexpression of EZH2 in cancer trimethylates H3K27 to inhibit gene expression, especially among tumor suppressor genes. (c) Cyclin-dependent kinase 1/2 phophorylates EZH2 at Th350 which results in deregulating tumor suppressor genes by increasing H3K27-trimethylation levels at promoters of EZH2 targeted genes. (d) Proposed anti-tumor mechanisms of action of CDK1/2 and EZH2/Th350 inhibitors.

Polycomb group (PcG) proteins are transcriptional repressors that inhibit developmental regulators in embryonic stem cells and silence tumor suppressor genes in cancer [80]. Enhancer of zeste homolog 2 (EZH2) is a subunit of the Polycomb-repressive complex 2 (PRC2), which catalyses the trimethylation of histone H3 on Lys 27 (H3K27) and is involved in genes repression. EZH2 is amplified and overexpressed in prostate cancer, with moderate increases in localized tumors, and higher expression in metastatic prostate cancers. Overexpression of EZH2 has been associated with the invasion and progression of prostate cancer [81, 82]. EZH2 is thought to promote tumorigenesis via epigenetic silencing of a group of tumor suppressor genes, including *ADRB2*, *CDH1*, *PSP94*, and *DAB2IP.* Overexpression of EZH2 trimethylates H3K27 and thus inhibits gene expression, particularly among tumor suppression genes (Figure 2).* DAB2IP* is a novel GTPase-activating protein for modulating the Ras-mediated signal pathway and tumor necrosis factor- (TNF-) associated apoptosis. The loss of *DAB2IP* expression is frequently detected in metastatic prostate cancer [83]. Epigenetic silencing of *DAB2IP* is a key mechanism by which the *EZH2* activates Ras and NF-*κ*B and triggers metastasis [84, 85]. 

Through genome-wide location analysis of prostate cancer cells, Yu et al. identified *SLIT2 *as a top target gene of EZH2-mediated H3K27 trimethylation. Overexpression of SLIT2 inhibits prostate cancer cell proliferation and invasion. The EZH2-containing Polycomb repressive complexes bound to the *SLIT2* promoter inhibiting its expression. SLIT2 was downregulated in a majority of metastatic prostate tumors exhibiting a negative correlation with EZH2. This repressed expression could be restored by methylation inhibitors or EZH2-suppressing compounds [86].

Recently, ETS transcription factors have emerged as important elements in prostate tumorigenesis due to the finding of recurrent translocations involving ETS genes, the most frequent being the TMPRSS2 : ERG gene fusion leading to overexpression of full length ERG [87]. Kunderfranco et al. performed a comprehensive analysis of the ETS gene family in prostatic normal and tumor tissues and established that the Polycomb group (PcG) protein EZH2 is a direct target of ERG and ESE3 and a key player in transcriptional silencing of the prostate-specific tumor suppressor gene Nkx3.1 [88].

## 6. Methylation as a Diagnostic and Prognostic Marker for Prostate Cancer

Recent studies have shown that methylation of selected genes may be useful as a biomarker for prostate cancer. *GSTP1* methylation appears to discriminate between benign and premalignant/malignant prostate and persists through all stages of prostate cancer, and can be detected in circulating tumor cells (CTCs) [29–32]. Methylation of *RASSF1*, *GSTP1*, *RAR*β**, and cadherin genes correlates with clinicopathological features of poor prognosis [14]. Methylation of *APC*, *cyclin D2*, *GPR7*, *ABHD9*, and expressed sequence tag on chromosome 3 (Ch3-EST) has been shown to be associated with Gleason score, pathological stage, and PSA recurrence [89–91].

## 7. Histone Modification as a Diagnostic and Prognostic Marker for Prostate Cancer

Histone modification patterns have similarly been found to predict risk of prostate cancer recurrence [76]. Overexpression of HDAC1 and HDAC2 conveys poor prognosis and has a highly significant negative PSA relapse-free survival [92, 93]. EZH2 is overexpressed in metastatic prostate cancer and is a marker of aggressive diseases. By stepwise cross-validation, Yu et al. developed a “Polycomb repression signature” composed of 14 direct targets of PcG in metastatic tumors. Prostate cancers in which this gene signature is repressed show poor clinical outcome and are associated with cancer progression [94]. SLIT2 is downregulated in prostate cancer by epigenetic mechanisms and represents a potent prognostic biomarker that merits further evaluation in large patient cohorts [86].

## 8. Epigenome-Targeted Therapy

### 8.1. Hypomethylating Agents in Prostate Cancer

In preclinical studies, the hypomethylating drug, 5-azacitidine (5-Aza), demonstrated synergistic effects with docetaxel and cisplatin in AR-positive 22RV1 and in AR-negative PC3 cells [95]. A structurally similar hypomethylating agent, decitabine (DAC), also exhibited synergy with cisplatin and cyclophosphamide in cell lines although the relationship to DNA hypomethylation as the mechanism was unclear [96]. 

In a phase II trial of single agent subcutaneous (SC) 5-Aza in 36 chemonaïve patients with progressive metastatic or nonmetastatic CRPC and PSA doubling times (PSAdt) ≤3 months, Sonpavde et al. demonstrated promising effects on PSA kinetics [97]. PSAdt was calculated over a period of 4 weeks at baseline and on therapy. A rapid PSAdt was chosen to enhance the detection of therapy-related changes in PSA kinetics; additionally, it is typical for metastatic CRPC to have a rapid PSA doubling time of <3 months. 5-Aza was administered at 75 mg/m^2^ SC for 5 days every 4 weeks for up to 12 cycles. One of the biological concepts was to resensitize the tumor to combination androgen deprivation therapy. Thus, LHRH agonist and antiandrogen were continued without antiandrogen withdrawal. Thirty-four of 36 enrolled patients were evaluable (81% with metastatic disease). A PSAdt of ≥3 months was attained in 19 patients (55.8%). Overall median PSAdt was significantly prolonged compared to baseline (2.8 versus 1.5 months, *P* < 0.01). Fourteen patients had some PSA decline during therapy and 1 patient had a ≥30% decline compared with baseline. The median clinical progression-free survival was 12.4 weeks. A phase I/II trial of 5-Aza with docetaxel and prednisone in metastatic CRPC progressing postdocetaxel is currently enrolling patients at the University of Miami (NCT00503984). The primary endpoint for the phase II portion of the trial is response by PSA or RECIST criteria. Correlatives planned include pre- and post-treatment methylation of DNA repetitive elements in peripheral blood mononuclear cells, GADD45*α* methylation in serum DNA, and optional prostate biopsy tissues using bisulfite treatment methylation assays [23]. Thus far, the clinical efficacy outcomes of 5-AZA in human prostate cancer trials have provided a hint of activity, but no overwhelming results. One possible reason is the instability of DNA methylation inhibitors in physiological conditions in that they became undetectable within a short time after administration [98]. This can lead cancer cells to take advantage of DNA methylation recovery systems, resulting in resilencing of DNA hypermethylated genes. Wong et al. provided strong evidence for DNA methylation recovery and found that H3K9 trimethylation and H3K27 trimethylation were closely associated with DNA methylation recovery [11]. In this regard, the efficacy of DNA methylation inhibitors in cancer treatment could be significantly improved if the DNA methylation recovery system could be suppressed or minimized.

### 8.2. HDAC Inhibitors in Prostate Cancer

Histone deacetylase (HDAC) is recognized as one of the promising targets for cancer therapy. In preclinical studies, Valproic acid inhibits growth of prostate cancer cells in vitro and reduces tumor xenograft growth in athymic nude mice owing to inhibition of histone acetylation by HDAC1. This agent has multiple effects, including cell-cycle arrest, increased apoptosis, decreased angiogenesis, and induction of senescence [2]. Vorinostat suppresses the growth of the LNCaP and PC-3 cell lines. Furthermore, it also shrinks tumors and suppresses their growth in mice transplanted with CWR22 human prostate tumor cells [99]. Romidepsin inhibits cell proliferation by arresting cell-cycle transition at the G1 and G2/M phases [100]. Entinostat arrests the growth of PC-3 and LNCaP cells in vitro, induces cell death in DU145 cells, and inhibits the growth of subcutaneous tumor xenografts of these three cell lines in vivo. Molecular analysis showed increased histone H3 acetylation and cyclin-dependent kinase inhibitor 1 (p21) expression in tumor samples from entinostat-treated patients. In the transgenic adenocarcinoma of the mouse prostate (*TRAMP*) model, long-term treatment with Entinostat slowed tumor progression and greatly reduced cell proliferation [2].

HDAC inhibitors have been noted to have greater antiproliferative effects on AR-positive prostate cancer cells than their AR-negative counterparts and inhibit xenograft growth in both castration-sensitive- and resistant models [99, 101]. In a study by Liu et al. LBH589 (Panobinostat) reversed the resistance of androgen-independent (AI) LNCaP cells to bicalutamide and to apoptosis. Treatment of bicalutamide-resistant AI cells with LBH589 combined with bicalutamide synergistically inhibited cell growth and induced a fivefold higher level of caspase 3/7 activation [102]. Proposed mechanisms of HDAC inhibitor clinical activity in prostate cancer include: preferential targeting of HDAC6 which deacetylates HSP90 and decreases AR stability, direct suppression of AR transcription, and sensitization of prostate cancer cells to DNA-damaging agents by targeting Ku70 acetylation [101, 103–105]. In light of their high potency to inhibit tumor cell growth in vivo, HDAC inhibitors have entered human clinical trial development. 

Bradley et al. reported phase II results in 27 metastatic CRPC patients with progressive disease after one prior chemotherapy regimen utilizing the oral HDAC class 1 and 2 inhibitor vorinostat administered at a continuous dose of 400 mg once per day [106]. No PSA declines ≥50% were observed, with best objective response of stable disease seen in only 2 patients (7%). In addition, therapy was associated with considerable toxicity with 44% of patients experiencing grade 3 adverse events. All patients were taken off of study prior to 6 months from therapy initiation. A statistically significant association was observed between high posttreatment IL-6 levels and treatment-related toxicity.

Similarly, Molife et al. reported phase II results in 35 patients with chemo naïve metastatic CRPC utilizing the intravenous HDAC inhibitor romidepsin administered at a dose of 13 mg/m^2^ iv days 1, 8, 15 on a 4-week cycle [107]. The primary endpoint of the study was 6-month disease control rate defined as the percentage of patients at 6 months with RECIST complete response, partial response, or stable disease. According to this definition, a disease control rate of 5.7% (2 of 35 patients) was observed. Eleven patients (31%) had a best response of stable disease; however these were short lived with none meeting the 6-month duration necessary to qualify for the 6-month disease control endpoint. Two patients demonstrated a PSA decline ≥50% with an additional 1 patient showing a PSA decline >30%. Eleven patients (31%) discontinued therapy due to toxicity.

In addition, combination HDAC inhibitor therapy with oral panobinostat and front-line docetaxel chemotherapy has been investigated by Rathkopf et al. in a phase I study in 16 patients with metastatic CRPC [108]. Patients received either single-agent panobinostat 20 mg oral once daily on days 1, 3, 5, 8, 10, 12 on a 21-day cycle or panobinostat 15 mg according to the same schedule in combination with intravenous docetaxel 75 mg/m^2^ on day 1. Both the single agent and docetaxel combination regimens were deemed feasible from a toxicity standpoint. No responses were seen with oral panobinostat alone. Five of eight patients (63%) on the panobinostat plus docetaxel arm demonstrated >50% PSA declines. In 9 of 11 patients, a >2-fold increase in peripheral blood mononuclear cell histone acetylation was observed on day 5 of cycle 1. The study was stopped after 16 patients due to a more favorable pharmacokinetic profile with an intravenous formulation of panobinostat. 

It is not clear why outcomes from clinical trials of HDAC inhibitors in metastatic CRPC have not matched the promising preclinical activity and scientific rationale. Given the high toxicity seen in these trials leading to dose reductions, it is possible that suboptimal cell inhibitory plasma concentrations of the HDAC inhibitors may explain why less clinical activity was seen than expected. While HDAC inhibitors can lead to activation of several silenced genes, several studies have shown that about the same number of genes are upregulated as are downregulated by these epigenetic modifying agents [109]. Therefore clarification of which gene is critical for clinical efficacy requires further studies. Histone acetylation as a biomarker for predictive treatment outcome has been questioned and, while useful as a surrogate for HDAC inhibition, does not appear to reflect tumor response.

## 9. Conclusion

Prostate cancer is a disease driven by progressive genetic and epigenetic aberrations. DNA methylation and histone acetylation are intimately linked, so that global hypomethylation might be expected to lead to global alterations in the level of histone acetylation and vice versa. These rapidly emerging data strongly indicate that the entire epigenome is fundamentally disturbed in prostate cancer development and therefore represents a target for therapeutic development. Altered DNA methylation, changes in the expression of chromatin proteins, and posttranslational histone modifications can be used for prostate cancer detection and classification. The reversible nature of DNA methylation forms the basis of epigenetic cancer therapy. However, it has been reported that DNA remethylation and gene resilencing could occur after removal of demethylation treatment, and this may significantly hamper the therapeutic value of DNA methylation inhibitors. We need a better understanding of the pharmacodynamics and biomarkers that predict response to HDAC inhibitors in prostate cancer. Epigenetic targeted therapy is in an early stage of development. Both at the mechanistic level and at the clinical/therapeutic level, much remains to be learned. Progress in this area of cancer therapeutics is promising; however, it is also challenging.

##  Conflict of Interests

The authors declare that there is no conflict of interests.

## Figures and Tables

**Table 1:** Hypermethylated genes in prostate cancer.

DNA repair gene	
*GSTP1*	
*MGMT*	
Tumor-suppressor genes	
*APC*	
*RAR*β**	
*RASSF1*	
Hormone receptor genes	
*AR*	
*ESR1,2*	
Cell adhesion genes	
*CDH1*	
*CDH13*	
*CD44*	
Cell-cycle control genes	
*CCND2*	
*CDKN1B*	
*SFN*	
Apoptotic genes	
*GADD45a*	
*PYCARD*	
*RPRM*	
*GLIPR1*	
